# Thermochemical Mechanism of the Epoxy-Glutamic Acid Reaction with Sn-3.0 Ag-0.5 Cu Solder Powder for Electrical Joining

**DOI:** 10.3390/polym13060957

**Published:** 2021-03-20

**Authors:** Gwang-Mun Choi, Ki-Seok Jang, Kwang-Seong Choi, Jiho Joo, Ho-Gyeong Yun, Chanmi Lee, Yong-Sung Eom

**Affiliations:** ICT Creative Research Laboratory/Materials & Components Research Division/Emerging Materials Research Section at Electronics and Telecommunications Research Institute (ETRI), 218 Gajeong-ro, Yuseong-gu, Daejeon 34129, Korea; sroka80@etri.re.kr (K.-S.J.); kschoi@etri.re.kr (K.-S.C.); jihojoo@etri.re.kr (J.J.); yunhg@etri.re.kr (H.-G.Y.); hichanmi@etri.re.kr (C.L.); yseom@etri.re.kr (Y.-S.E.)

**Keywords:** epoxy-based solder paste, l-glutamic acid, tin carboxylate salt, etherification, electrical joining

## Abstract

An epoxy-based solder paste (ESP) is a promising alternative to conventional solder pastes to improve the reliability of fine-pitch electrical joining because the epoxy encapsulates the solder joint. However, development of an appropriate epoxy formulation and investigation of its reaction mechanism with solder powder is challenging. In this study, we demonstrate a newly designed ESP consisting of diglycidyl ether of bisphenol F (DGEBF) resin, Sn-3.0 Ag-0.5 Cu (SAC305) solder powder, and L-glutamic acid (Glu), which is a proteinogenic amino acid for biosynthesis of proteins in living systems. The mechanism of the thermochemical reaction was explored and tentatively proposed, which reveals that the products of the reaction between SAC305 and Glu function as catalysts for the etherification of epoxides and alcohols produced by chemical bonding between DGEBF and Glu, consequently leading to highly crosslinked polymeric networks and an enhancement of impact resistance. Our findings provide further insight into the mechanism of the reaction between various formulations comprising an epoxy, amino acid, and solder powder, and their potential use as ESPs for electrical joining.

## 1. Introduction

Solder paste, mainly consisting of a solder alloy, a flux, an activator, and a solvent, plays an important role as an electrical joining material in the field of surface mount technology of electronic packaging for interconnection between semiconductor chips and substrates [1]. However, ongoing miniaturization of electronic components such as mini-LEDs, which range in size from 100 to 200 μm and small-sized multilayer ceramic capacitors (MLCCs), has presented considerable technical issues due to the need for new electrical bonding materials [2]. The decrease in chip size has led to a decrease in the joint area, which is largely related to the bonding force for mechanical reliability of the device. In addition, the cleaning process, which is required to completely remove the flux residue for corrosion prevention after the solder reflow bonding process, has become more challenging because of fine-pitch interconnections [3]. Further, generation of harmful fumes during solder reflow, which can be mainly ascribed to the flux and solvent, does not conform to the emerging requirement for eco-friendly manufacturing processes [4].

Over the last few decades, epoxy-based solder pastes (ESPs) have generated considerable interest as a leading option to overcome the disadvantages of commercial solder pastes. Eom et al. have conducted extensive research on the mechanism of the reaction between epoxy-based resins and solders [5,6,7,8] and their thermomechanical and electrical properties for their application as conductive adhesives for electronic packaging [9,10,11,12,13]. The authors also proposed a working mechanism for a formulation consisting of epoxy resins, curing agents, catalysts, reductants such as carboxylic acids, and solder powder [9,10]. According to this mechanism, the oxidized surface layer of the solder powder is rapidly eliminated by the activated carboxylic acid groups in the reductant before reaching the reflow soldering temperature. Above the liquidous temperature of the solder alloy, the molten solder particles start to agglomerate, and the resin formulation is pushed away simultaneously. After the formation of the solder joint, the epoxy-based resin is cured, providing reliable physical, chemical, and electrical protection. The enhancement effect of the ESP on the lifetime of solder joints has been clearly recognized by thermomechanical reliability analysis [14,15]. Fukumoto et al. introduced thermoplastic polyester resins into ESPs consisting of epoxy-based thermoset resins, curing agents, and diethyl glutaric acid as the reducing agent for self-organization soldering [16] and prevention of spreading of the ESP [17]. S.-B. Jung et al. reported the composition of reaction products produced after reflow tests of an ESP comprising epoxy resins, a succinic acid-based flux as the reductant, and Sn-58 Bi solder powder [18]. A review by J.P. Jung et al. widely covers the characteristics, advantages, and disadvantages of ESPs compared with the conventional solder pastes and potential issues in electronic packaging applications [19]. However, only a few studies have focused on unveiling the thermochemical mechanism of the reactions between the constituents of ESPs [7,8,18]. Particularly, the function of the product formed in the reaction between solder oxides and reductants remains unclear. For commercialization and expansion of the application range of ESPs, an in-depth understanding of the reaction mechanism and novel approaches for epoxy formulation are required.

Amino acids, which contain an amine, a carboxylic acid, and a side chain attached to the alpha (α)-carbon, are biochemical building blocks for a vast array of proteins in living systems [20]. Owing to the coexistence of amine and carboxylic acid groups in one molecule, the reaction of epoxy with amino acids such as lysine [21] and tryptophan [22] as curing agents were studied. Some amino acids have been occasionally used as components of organic fluxes to dissolve the solder oxides in the solder paste industry [23]. Inspired by the above-mentioned studies, we decided to use amino acids as both curing and reducing agents for the ESP. In this study, glutamic acid (Glu) was chosen among the 20 standard amino acids because it is the most produced species [24] and contains a gamma (γ)-carboxylic acid as a side chain that can act as an additional reducing agent. In the case of epoxy resins for the ESP, it is important to use an epoxy with low viscosity to improve solderability by providing sufficient mobility to solder particles [17]. Therefore, diglycidyl ether of bisphenol F (DGEBF), which is readily available and shows a relatively low viscosity of about 3000 cPs at 25 °C, was used. Herein, the thermochemical reactions between DGEBF, Glu, and Sn-3.0 Ag-0.5 Cu (SAC305) solder powder were studied. In particular, the effect of the products formed by the reaction between Glu and SAC305 on the curing behavior of DGEBF with Glu was thoroughly investigated and a plausible mechanism was proposed. Thus, a newly designed ESP comprising epoxy, Glu, and SAC305 was developed for electrical joining.

## 2. Materials and Methods

### 2.1. Materials

Diglycidyl ether of bisphenol F (DGEBF), which corresponds to ~170 g/epoxy equivalents, was supplied by Kukdo Chemical (Seoul, Korea). l-glutamic acid (Glu, ≥99%) and 2-butanone (≥99%) were purchased from Sigma-Aldrich (St. Louis, Missouri, MO, USA). SAC305 type-6 solder powder, of which at least 80% of the particles met the size requirement of 5–15 μm in accordance with IPC standard J-STD 005 [25], was procured from Alpha Metals (South Plainfield, New Jersey, NJ, USA). The oxygen concentration of SAC305 type-6 solder powder was measured by the TC500 nitrogen/oxygen determinator (Leco Corporation, St. Joseph, Michigan, MI, USA). For comparative study, a conventional solder paste consisting of SAC305 type-6 solder powder (88 wt%) and flux-based composition with solvents (12 wt%) was purchased from Hojeonable (Daejeon, Korea). All materials were used as received without any further purification.

### 2.2. Formulation Preparation 

All formulations in this work were prepared as follows. Four mixtures of DGEBF and Glu with different equivalents (*x* = molar ratio of epoxy to Glu) were prepared by mixing DGEBF and Glu at weight ratios of 2.311:1 (*x* = 2), 3.466:1 (*x* = 3), 4.622:1 (*x* = 4), and 5.777:1 (*x* = 5). The DGEBF/Glu mixture with *x* = 4 was designated as epoxy-Glu formulation (EG). In addition, two formulations of DGEBF, Glu, and SAC305 were prepared by mixing EG and the SAC305 type-6 solder powder at weight ratios of 99:1 and 20:80, which were termed EG with 1 wt% of SAC305 formulation (EGS-1) and EG with 80 wt% of SAC305 formulation (EGS-80), respectively. A roll mill machine and a paste mixer were used to prepare the above-mentioned formulations before characterization.

### 2.3. Differential Scanning Calorimetry (DSC) Analysis

The DSC experiments were performed using a DSC Q20 calorimeter (TA instruments) under nitrogen (N_2_). Hermetic aluminum pans were used as containers for reference and samples (~5 mg). In the case of the dynamic DSC, a heating rate of 10 °C/min was used from −60 to 300 °C. For isothermal DSC experiments, the following temperature profile was used: fast heating from 25 °C to the target temperatures (180 or 240 °C) at a heating rate of 120 °C/min and holding at the target temperatures for 30 min. 

### 2.4. Thermogravimetric Analysis (TGA)

To evaluate thermal decomposition behavior, TGA experiments were carried out using a TGA Q50 thermogravimetric analyzer (TA instruments, New Castle, Delaware, DE USA). All samples (~15 mg) were loaded into open aluminum pans used as disposable sample containers. In the case of the dynamic TGA, the samples were heated from room temperature up to 350 °C with a heating rate of 10 °C/min under an N_2_ atmosphere of 50 mL/min. Isothermal TGA was conducted by heating from room temperature to 240 °C at a heating rate of 120 °C/min and holding at the temperature for 10 min under N_2_ condition. 

### 2.5. Fourier-Transform Infrared (FTIR) Analysis

To study the thermochemical reactions between DGEBF, Glu, and SAC305, EG and EGS-1 were thermally cured under three different temperature conditions: 180, 210, and 240 °C for 30 min. The cured samples of EG and EGS-1 at 240 °C for 30 min were termed c-EG and c-EGS-1, respectively. The FTIR spectra (4000–400 cm^–1^) were obtained before and after the thermal curing experiments of EG and EGS-1 in an attenuated total reflection (ATR) mode using a Nicolet iS50 analytical FTIR spectrometer equipped with a built-in diamond ATR module (Thermo Fisher Scientific, Waltham, Massachusetts, MA, USA).

### 2.6. Dissolution Test

Polymer dissolution experiments were examined by immersion of c-EG and c-EGS-1 samples in 2-butanone for 24 h at room temperature. The solubility was quantitatively checked by measuring the weight before and immediately after immersion and after drying for 1 day at 60 °C. 

### 2.7. High-Temperature Observation Test

High-temperature behaviors of c-EG and c-EGS-1 were visually recorded using a SMT Scope SK-5000 (Sanyo Seiko, Tokyo, Japan). The samples were placed in the chamber and heated from room temperature to 240 °C at a heating rate of 120 °C/min.

### 2.8. Mechanical Characterization

To characterize the mechanical performance of c-EG and c-EGS-1, Berkovich nanoindentation experiments were performed at a constant strain rate of 0.05 s^−1^ up to a maximum load of 200 mN using an iMicro nanomechanical test system equipped with an InForce 1000 transducer and an optical microscope. Bulk samples (~1 mm thickness) were used to extract intrinsic properties without any interference effect of the substrate. Assuming that the Poisson ratio of all samples was 0.3, elastic modulus and hardness values were obtained using the continuous stiffness measurement (CSM) technique with an oscillation frequency of 110 Hz according to the nanoindentation loading depth. In addition, nanoindentation creep tests were executed: fast loading from 0 to 200 mN at a constant strain rate of 1 s^−1^, holding at the maximum load for 300 s followed by unloading. Furthermore, impact resistance was evaluated using a custom-made ball drop tester with a steel ball of 45 g and a height of 50 cm. For comparison, we used samples of approximately the same size (~5 × 5 × 1 mm^3^).

### 2.9. Viscosity Measurement

Viscosity was measured at 10 rpm and 25 °C using a Brookfield rotational viscometer (HBDV-II+Pro) equipped with a small chamber adaptor. The spindle and sample chamber were SC4–14 and SC4-6RP, respectively. To minimize the effect of uneven temperature, the viscosity measurement was conducted 30 min after the sample was loaded into the chamber kept at 25 °C.

### 2.10. Reflow Test

To transfer EGS-80 and the conventional solder paste on test boards, a screen printing process was performed using a 30-µm-thick metal mask. Thermal reflow behaviors of the samples were visually captured using the SMT Scope SK-5000 (Sanyo Seiko, Tokyo, Japan) with the following reflow temperature profile under N_2_ condition: heating from room temperature up to 240 °C at a heating rate of 120 °C/min, holding at 240 °C for 10 min, and cooling to room temperature with a nominal cooling rate of 120 °C/min.

## 3. Results and Discussion

**Figure 1:**
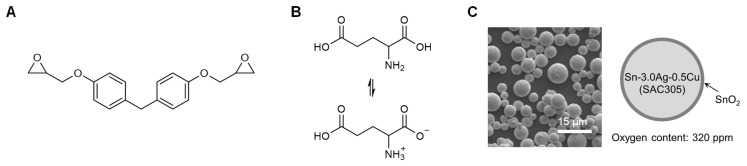
Materials used in the study. (**A**,**B**) Chemical structure of diglycidyl ether of bisphenol (DGEBF) (**A**) and Glu (**B**). (**C**) SEM image (left) and illustration (right) of SAC305 type-6 powder.

Figure 1 shows the materials used in this study. The amino acid group of Glu is known to exist in two electrically neutral forms, i.e., an uncharged state and the more preferential form at room temperature, a zwitterion (Figure 1B) [26]. We attempted to assess whether Glu could function as a curing agent for DGEBF as well as a reductant for the oxide layer of SAC305. The degree of oxidation of SAC305 type-6 particles was confirmed by measuring the oxygen concentration, which was found to be 320 ppm.

**Figure 2:**
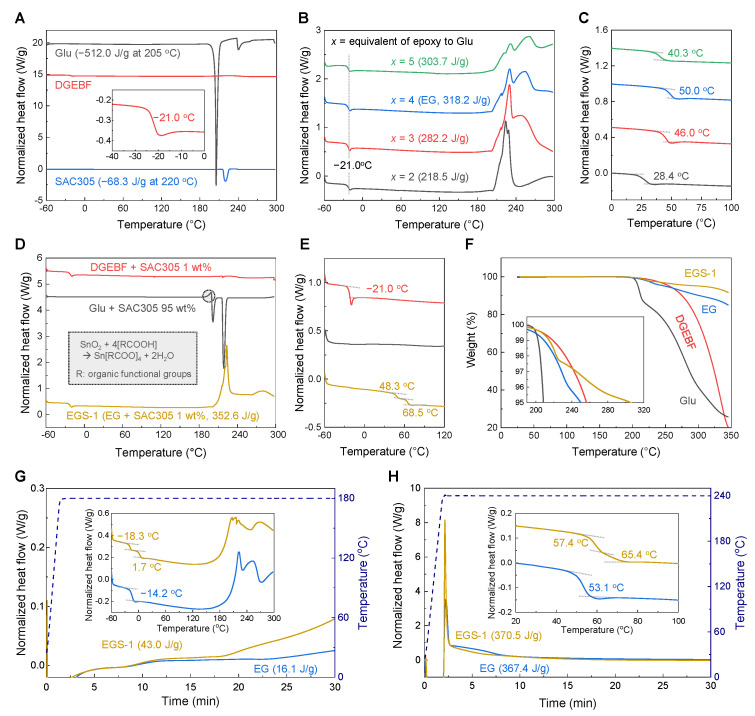
Thermal reaction between DGEBF, Glu, and SAC305. (**A**) Dynamic DSC results of Glu, DGEBF, and SAC305. The inset graph is an expanded curve of DGEBF, exhibiting uncured *T_g_*. (**B**,**C**) 1st dynamic (**B**) and 2nd dynamic (**C**) DSC curves between DGEBF and Glu with different equivalent ratios (*x*). The mixture of DGEBF and Glu with *x* = 4 was deemed to be optimal and abbreviated as EG. (**D**,**E**) 1st dynamic (**D**) and 2nd dynamic (**E**) DSC curves of DGEBF/Glu/EG with SAC305. Inset chemical reaction (**D**) is related to the marked exothermic reaction. EG with 1 wt% of SAC305 was designated as EGS-1. (**F**) Dynamic TGA results of DGEBF, Glu, EG, and EGS-1. The inset shows an expanded high-temperature region. (**G**,**H**) Isothermal DSC results of EG and EGS-1 at 180 °C (**G**) and 240 °C (**H**) for 30 min. The inset curves exhibit 2nd dynamic DSC scans after the corresponding isothermal ones.

The thermochemical reaction between DGEBF, Glu, and SAC305 was studied using DSC and TGA. Figure 2A shows the dynamic DSC curves of individual reactant materials. There was no notable effect of increasing temperature on DGEBF and SAC305 except for glass transition at −21 °C [27] and melting at 217 °C [28], respectively. However, Glu exhibited a huge endothermic reaction at 205 °C, which corresponded to thermal decomposition and lactam transformation [20]. Based on the knowledge of possible reactions of epoxy with carboxylic acid and the amine in Glu [29], we evaluated the curing behavior of DGEBF and Glu according to the different equivalents (*x*: moles of epoxy that could be reacted with one mole of Glu). Interestingly, all samples showed exothermic heat flow instead of an endothermic flow at ~205 °C (Figure 2B), indicating that the formation of chemical bonds between DGEBF and Glu suppressed the decomposition and internal cyclization of Glu. Based on quantitative measurements, the mixture with *x* = 4 released the highest exothermic energy of 318 J/g (Figure 2B) and the glass transition temperature (*T_g_*) of the cured sample obtained from the 2nd dynamic DSC scan was 50 °C (Figure 2C). This may be explained by the fact that Glu has four reactive groups for epoxy; epoxy can be esterified with the two carboxylic acids, one attached to the side chain and one at α-carbon, and the two active hydrogens of the α-amino group that allow amine-epoxy addition. Therefore, the formulation of DGEBF and Glu with *x* = 4 (EG) was optimal. Next, the thermal reactivity of SAC305 to DGEBF, Glu, and EG was examined by dynamic DSC analysis (Figure 2D,E).

There was no noticeable change in heat flow during the combination of DGEBF and SAC305, and *T_g_* of the epoxy was maintained at −21 °C before and after DSC heating. However, a sample of Glu with SAC305 exhibited a distinct exothermic peak at ~200 °C, which could not be identified in each material. It is generally accepted that the unexpected heat flow is due to the reaction of carboxylic acids with tin oxides at the surface of SAC305 particles leading to a generation of tin carboxylate salts [30], as described in the inset of Figure 2D. Interestingly, this reaction seems to be related to the catalytic effect of SAC305 on the curing behavior of EG, as confirmed by the dynamic DSC scan of EG with 1 wt% of SAC305 (EGS-1) in Figure 2D exhibiting a shift of the exothermic reaction toward low temperature compared with that of EG alone (Figure 2B) as well as an increase in reaction heat from 318 to 353 J/g. In addition, two glass transitions were observed in the 2nd dynamic DSC result of ESG-1 (Figure 2E), i.e., *T_g_* = 48 °C, which was comparable to that of the 2nd dynamic DSC of EG (Figure 2C), and the higher *T_g_* of 69 °C. This result likely implies that the tin carboxylate salts, which are the products of the reaction between Glu and SAC305, function as a catalyst for chemical reactions such as etherification rather than assisting in the formation of chemical bonds of epoxides with carboxylic acids and amines [31]. To investigate the thermal stability of DGEBF, Glu, EG, and EGS-1, we performed dynamic TGA under N_2_ flowing condition (Figure 2F). While thermal decomposition of Glu was significantly reduced by its reaction with DGEBF, EGS-1 showed the highest 5% weight loss temperature of 305 °C, which indicated that SAC305 contributed to the formation of cross-linked polymeric networks in the EG matrix. 

The effect of SAC305 on the curing behavior of EG according to temperature was evaluated by conducting isothermal DSC experiments at 180 and 240 °C for 30 min. Although the reactions of EG and EGS-1 at 180 °C proceeded quite slowly (Figure 2G) in accordance with the inherent low reactivity of amino acid groups [22], the addition of SAC305 slightly increased the reaction rate leading to an increase in exothermic heat from 16 to 43 J/g and the formation of polymeric species with a higher *T_g_* of 2 °C. In the case of isothermal DSC conducted at 240 °C (Figure 2H), EG reacted completely with one sharp peak followed by a broad shoulder. The sharp peak became stronger with a lowered shoulder reaction in EGS-1, which resulted in higher cured *T_g_* values of 57 and 65 °C as compared to those of EG. It is noteworthy that the low reactivity of amino acids because of their electrostatic interaction was activated only when sufficient heat was applied, even though the tin carboxylate salts were created and participated as a catalyst in the early stages of the reaction. This seems to imply that the tin carboxylate salt did not participate in the reaction between DGEBF and the amino acid groups in Glu.

**Figure 3:**
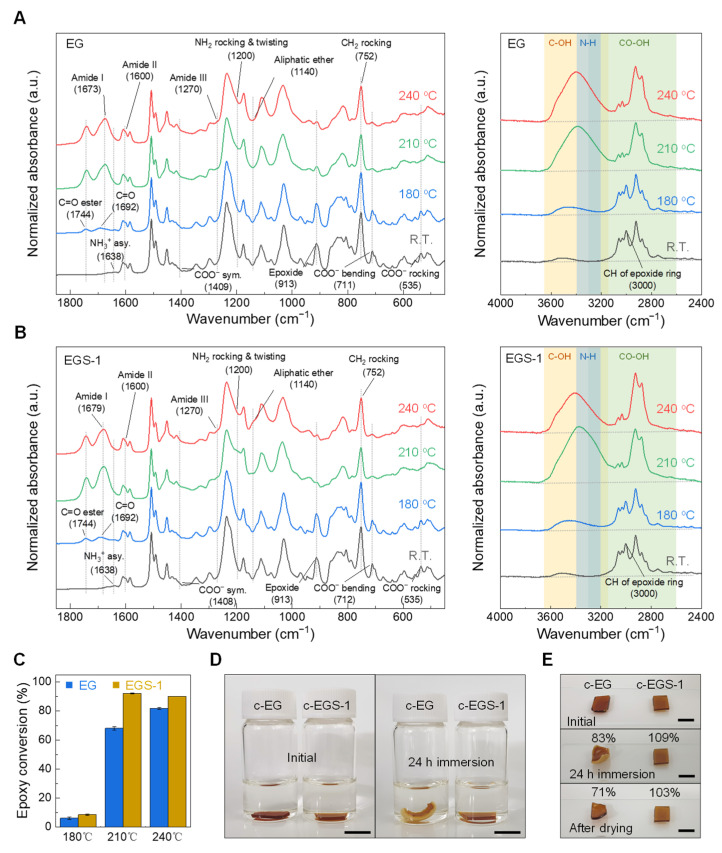
Crosslinking effect of SAC305 on the thermochemical reaction of DGEBF with Glu. (**A**,**B**) Representative low wavenumber (left) and high wavenumber (right) diamond ATR-FTIR spectra of EG (**A**) and EGS-1 (**B**) at room temperature, and after thermal reaction at 180/210/240 °C for 30 min, respectively. Owing to the broad absorption of diamond at ~2000 cm^−1^, data in the range of 1900 to 2400 cm^−1^ were not considered. Gray dashed lines and shaded areas were used to indicate the changes in corresponding peaks. (**C**) Epoxy conversions of EG and EGS-1 after curing at 180/210/240 °C for 30 min, which were calculated from the corresponding FTIR spectra. Data are presented as mean ± SD, *N* = 5. The cured samples of EG and EGS-1 at 240 °C for 30 min are abbreviated as c-EG and c-EGS-1, respectively. (**D**) Photographs of c-EG and c-EGS-1 after immersion in 2-butanone for 0 h (left) and 24 h (right); scale bar, 0.5 cm. (**E**) Photographs of c-EG and c-EGS-1 before (top), immediately after the 24 h immersion test (middle), and after drying (bottom). Percentage values show the change in weight compared to the initial state; scale bar, 0.5 cm.

For better understanding of the mechanism of the thermochemical reaction between DGEBF, Glu, and SAC305, FTIR analysis was performed on EG and EGS-1 before and after thermal curing at 180, 210, and 240 °C for 30 min. Figure 3A,B shows the changes in the FTIR spectra of EG and EGS-1, respectively, according to the curing temperatures. The corresponding epoxy conversions (Figure 3C) were calculated using the following Equation:

(1) $$D_{ec}(\%)=\left(1-\frac{A_{E}\prime }{A_{E}}\right)\times 100$$

where *A_E_* and *A_E_*′ are normalized areas of the epoxy group peak at 913 cm^−1^ with the CH_2_ peak at 752 cm^−1^ as a reference in the FTIR spectra obtained before and after the curing process, respectively [32]. At room temperature, EG and EGS-1 showed nearly identical characteristic peaks, confirming the zwitterionic nature of amino acids such as the asymmetric deformation of NH_3_^+^ at 1638 cm^−1^ and symmetric stretching, bending, and rocking vibrations of COO^−^ at 1408, 712, and 535 cm^−1^, respectively. Besides, a similar curve change was found in both samples after heating at 180 °C. The zwitterion peaks did not change appreciably, while several new peaks were observed, including two distinctive carbonyl stretching absorptions at 1744 and 1692 cm^−1^ with the hydroxyl of alcohol ranging from 3100 to 3700 cm^−1^ [33]. The appearance of the carbonyl stretching found at a high wavenumber and hydroxyl group absorptions were attributed to the esterification of the epoxide with γ-carboxylic acids as most of amino acids exist as inactive species at 180 °C [22]. The C=O stretch at 1692 cm^−1^ likely corresponds to amide I of the 5-membered lactam bonds produced by the internal cyclization of Glu with a few γ-carboxylic acids that did not react with epoxy groups, which is a well-known transformation of Glu under high temperature [20,34]. The epoxy conversion of EGS-1 at 180 °C was slightly higher than that of EG. This result was attributed to the catalytic effect of tin carboxylate salts generated by the reaction of γ-carboxylic acids and tin oxides [30], which is supported well by the isothermal DSC results (Figure 2G).

After the curing of EG at 210 °C, the characteristic peaks of the zwitterion disappeared and the intensity of the epoxy peaks at 913 and 3000 cm^−1^ decreased. The epoxy conversion was 68.2%. Further, the intensity of peaks corresponding to carbonyl stretching of ester, alcohol, and amide (I) (mainly C=O stretch, 1673 cm^−1^), (II) (N-H bend with C-N stretch, 1600 cm^−1^), and (III) (C-N stretch with N-H bend, 1270 cm^−1^) increased [35], although amide II was not well identified owing to overlap with the C-C stretching region for the aromatic ring of DGEBF [32]. These observations suggest that the zwitterions were converted into the uncharged normal form with sufficient heat energy [26] leading to participation of α-carboxylic acids and amino groups in the following reactions: peptide bond formation via dehydration between two amino acids as confirmed by increased intensity of amide I, II and III bands; reaction of epoxy with γ- and α-carboxylic acids and amine, resulting in a decrease in the epoxy peak height with enhanced peak strength of carbonyl ester and alcohol. While EGS-1 also cured at 210 °C, it exhibited completely different behavior. Although the zwitterions were similarly reduced, as confirmed by the decreased intensity of corresponding peaks, the epoxy conversion drastically increased up to 92.2% without adequate consumption of activated amino acids, as identified by the rocking and twisting of NH_2_ at 1200 cm^−1^ [36], amine stretching near 3300 cm^−1^, and O-H stretching of carboxylic acid in the broad region of 3300–2600 cm^−1^ [33]. The heights of the characteristic peaks of the peptide bond and aliphatic ether (1140 cm^−1^) were also noticeably increased [33]. It is reasonable to suppose that most of the epoxides were depleted by etherification owing to the catalytic effect of tin carboxylate salts [37], and the activated amino acids then reacted with each other to form peptide bonds while the unreacted species remained. When curing was carried out at 240 °C, there were no obvious peak changes between the two spectra at 210 and 240 °C in the case of EG, except for the increase in the epoxy conversion from 68.2% to 81.8%. On the other hand, EGS-1 cured at 240 °C showed reduced peak heights of the carboxylic acid and amine observed in the sample cured at 210 °C, which meant that the zwitterions were effectively activated by sufficient heat energy and participated in the reaction with epoxides before their exhaustion by etherification. Notably, the peak intensity related to the aliphatic ether in EGS-1 at 240 °C became much stronger with considerably reduced O-H stretching from 3100 to 3700 cm^−1^ even if the epoxy conversion was slightly lower as compared to that of EGS-1 at 210 °C. This phenomenon may be explained by the etherification between alcohols and the tin carboxylate salts acting as an acidic catalyst for this transformation at high temperature [38,39].

To investigate the formed polymeric structure in EG and EGS-1 cured at 240 °C for 30 min, termed as c-EG and c-EGS-1, respectively, the solubility of the samples in 2-butanone was tested (Figure 3D,E). After immersion, c-EG was completely swollen and warped by the solvent, similar to thermoplastics consisting of non-crosslinked polymeric networks [40]. In contrast, c-EGS-1 exhibited no visual change. Quantitatively, dissolution of about 30 wt% of c-EG was confirmed while c-EGS-1 showed a slight increase in weight because of absorption of the solvent that could not be completely removed after drying. This result is consistent with the observation that c-EGS-1 is composed of chemically crosslinked networks between DGEBF and Glu with the aid of SAC305, in contrast to the composition of EG. The completely different polymeric structures of both samples were also verified by high-temperature observation experiments (Videos S1 and S2), which clearly show the thermoplastic behavior of c-EG and the thermosetting nature of c-EGS-1.

**Figure 4:**
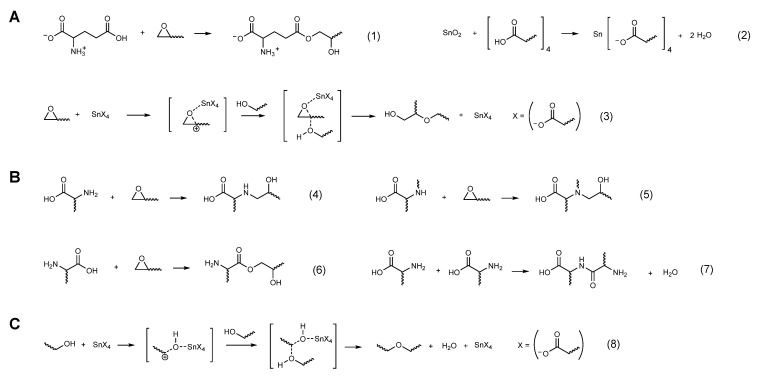
Proposed mechanism of thermochemical reaction between DGEBF, Glu, and SAC305. (**A**) Reaction of epoxy and γ-carboxylic acid of Glu with SAC305. Esterification of epoxy with γ-carboxylic acid (1). Dissolution of tin oxide by γ-carboxylic acid (2). Etherification between epoxy and hydroxyl groups with the catalytic effect of tin carboxylate salt (3). (**B**) Reaction of epoxy and activated amino acid group. α-Primary amine-epoxy addition (4). α-Secondary amine-epoxy addition (5). Esterification of epoxy with α-carboxylic acid (6). Peptide bond formation between two amino acid molecules (7). (**C**) Etherification between alcohols with the catalytic effect of a tin carboxylate salt (8).

Based on the above results, a tentative mechanism for the reaction between DGEBF, Glu, and SAC305 was proposed according to the temperature (Figure 4). In the first step, DGEBF and Glu are chemically linked by esterification of epoxides with γ-carboxylic acids (1). Simultaneously, tin oxides of SAC305 are dissolved by γ-carboxylic acids of Glu, leading to the formation of tin carboxylate salts (2), which may function as a Lewis acid and trigger a reaction between epoxides and alcohols that are generated by esterification (3). Above the critical temperature of ~205 °C, the reaction barrier due to the electrostatic hindrance of zwitterionic status of Glu almost disappears by its conversion into the uncharged neutral form. Thus, the α-primary amines react with epoxides (4), followed by α-secondary amine-epoxy addition reactions (5). Simultaneously, α-carboxylic acids are chemically linked with epoxides and α-primary amines attached to other α-carbons, resulting in the formation of esters (6) and peptide bonds (7), respectively. Further, a Lewis acid-catalyzed etherification of alcohols occurs at high temperature (8). The proposed mechanism is a preliminary attempt based on current results and might be proven incomplete. However, its significance is that the monomers of DGEBF and Glu can be thermally converted into a cured material that is highly crosslinked with ether bonds aided by the tin carboxylate salts, which are the reaction products of Glu and SAC305.

**Figure 5:**
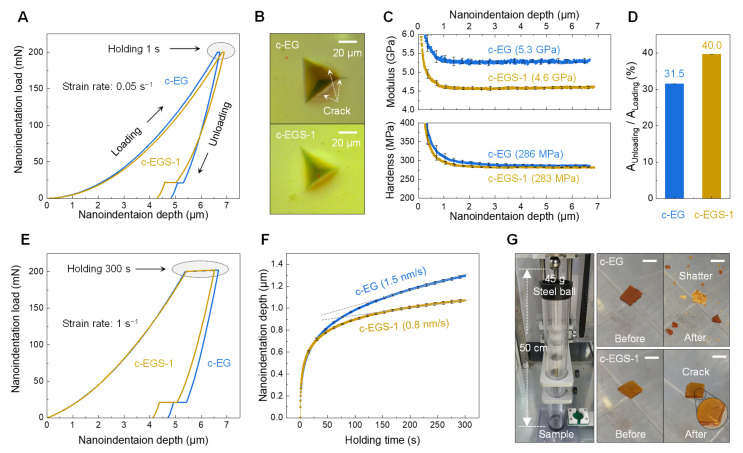
Mechanical characterization. (**A**) Representative nanoindentation load-depth curves with strain rate of 0.05 s^−1^ and 1 s holding at the maximum load. (**B**) Optical microscopy images exhibiting residual impression after the nanoindentation tests (**A**). (**C**) Nanoindentation modulus (top) and hardness (bottom) to depth curves of c-EG and c-EGS-1. Elastic modulus and hardness values were obtained at the nanoindentation depth of 5 µm. All data are presented as mean ± SD, *N* = 5. (**D**) Area ratio of unloading to loading curve in nanoindentation tests (**A**); mean ± SD, *N* = 5. (**E**) Representative nanoindentation load-depth curves with strain rate of 1 s^−1^ and 300 s holding at the maximum load. (**F**) Nanoindentation depth–time curves during the holding segments in the tests (**E**) for creep analysis. The gray dashed lines were used to show the slope of the creep rate of c-EG and c-EGS-1 in the steady-state creep region; mean ± SD, *N* = 5 (**G**) Photographs showing c-EG (top) and c-EGS-1 (bottom) before and after ball drop test (left); scale bar, 0.5 cm.

To evaluate mechanical responses according to their polymeric structures, especially with or without crosslinked networks, nanoindentation tests were performed with a Berkovich diamond tip on bulk samples of c-EG and c-EGS-1, as shown in Figure 5A. Compared with c-EG, a small hysteresis of the nanoindentation load to depth curves was observed in c-EGS-1 without radial and circumferential cracks around the residual impression (Figure 5B). In addition, elastic modulus (*E*) and hardness (*H*) values were obtained using the CSM method during the loading process. It should be noted that the ratio of *H* to *E* is closely related to elastic strain to failure [41]. The higher *H*/*E*, the stronger is the resistance to cracking. While both samples showed similar *H* values of ~285 MPa, a relatively low *E* value of 4.6 GPa was attained in c-EGS-1 despite the higher level of crosslinking, which led to a higher *H*/*E* value (0.06) than that of c-EG. This result proves that the two formulations were cured by different reaction mechanisms and that the relatively low *E* value of c-EGS-1 was due to an inherent flexible characteristic of the ether linkage [42]. Therefore, when an external force is applied, c-EGS-1 can be more easily deformed than c-EG and then elastically restored to its original shape, which is supported well by the higher area ratio of unloading to loading curve of c-EGS-1 (Figure 5D), which is used as a good indicator of elastic recovery [43]. To further investigate creep behavior of both samples, we conducted Berkovich nanoindentation creep tests as shown in Figure 5E, i.e., loading with a high strain rate of 1 s^−1^ followed by a holding segment for 300 s at the maximum load [44]. Figure 5F shows the nanoindentation depth vs. time curves during the corresponding holding segment. A comparatively low creep rate of 0.8 nm/s in c-EGS-1 was confirmed, which was attributed to its highly crosslinked polymeric networks. To check the mechanical performance under real application conditions, steel-ball drop tests were performed (Figure 5G, Videos S3 and S4). Upon impact, c-EG was completely shattered. On the other hand, a small crack was observed in c-EGS-1, revealing its potential use as an impact-resistant material.

**Figure 6:**
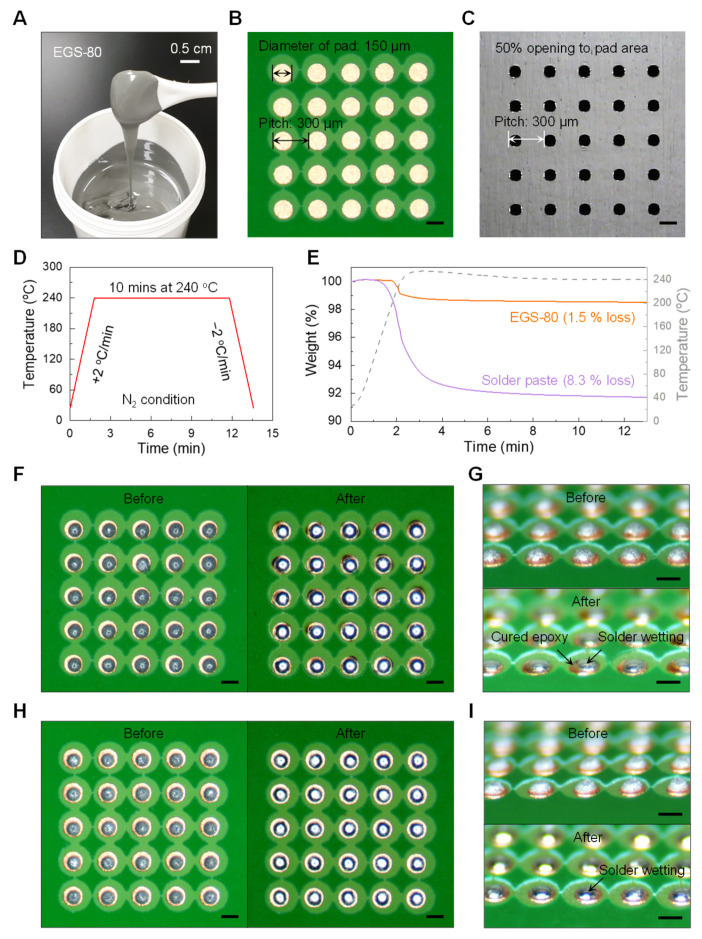
Potential use of EGS-80 as an epoxy-based solder paste (ESP). (**A**) Photograph of EGS-80. (**B**) Test board image with a 5 × 5 array of bare Cu pads; scale bar, 150 μm. (**C**) Metal mask image with 50% opening to the pad area of the test board (**B**); scale bar, 150 μm. (**D**) Thermal reflow profile condition. (**E**) Isothermal TGA results of EGS-80 and the solder paste at 240 °C for 10 min. The gray dashed line shows the actual temperature profile. (**F**) Top-view images of EGS-80 before (left) and after (right) the thermal reflow test (**D**); scale bar, 150 μm. (**G**) Side-view images of EGS-80 before (top) and after (bottom) the thermal reflow test (**D**); scale bar, 150 μm. (**H**) Top-view images of solder paste before (left) and after (right) the thermal reflow test (**D**); scale bar, 150 μm. (**I**) Side-view images of solder paste before (top) and after (bottom) the thermal reflow test (**D**); scale bar, 150 μm.

Based on chemical reactions between DGEBF, Glu, and SAC305, EG with 80 wt% of SAC305 (EGS-80) was formulated and evaluated for application as an ESP for isotropic electrical joining. Figure 6A shows the fabricated EGS-80, which had a viscosity of ~200,000 cPs at 25 °C, comparable to that of conventional solder pastes. For comparative study, EGS-80 and the solder paste (~190,000 cPs at 25 °C) were transferred on 5 × 5 target pads of the test board (Figure 6B) via screen printing using a metal mask with 50% openings in the pad area (Figure 6C) and thermally reflowed in accordance with the profile condition (Figure 6D), respectively. To evaluate outgassing characteristics during the reflow test, isothermal TGA experiments were performed under near identical conditions. As shown in Figure 6E, there was very little weight loss of EGS-80 as compared with that of the solder paste that contained volatile solvents and fluxes. In addition, Figure 6F,G and Video S5 reveal that EGS-80 maintained its shape without being pushed out of the pads below the liquidous temperature of SAC305 [45]. This characteristic is preferable because otherwise the solder particles may spread around the pads and lead to a short circuit between the joints. Once a temperature of ~219 °C was reached, SAC305 particles were reflowed, merged into one, and wetted well on the target pads without any satellite solder balls, which implied that the oxide layer of SAC305 particles was sufficiently removed by the carboxylic acids of Glu and the viscosity of the epoxy-based resin did not increase enough to limit the mobility of the molten solder particles as the epoxy curing reaction did not sufficiently proceed prior to solder wetting [7,17]. Thus, it was confirmed that EGS-80 showed almost similar solder wetting characteristics as the conventional solder paste (Figure 6H,I and Video S6). Furthermore, after the reflow test, the epoxy composition in EGS-80 was pushed around the solder joint and cured as shown in Figure 6G, in contrast to the conventional solder paste (Figure 6I). Therefore, EGS-80 can be expected to encapsulate the electrical joining and improve its mechanical reliability.

## 4. Conclusions

Herein, we studied the thermochemical reactions between DGEBF, Glu, and SAC305, which revealed that the number of reactive groups of Glu that participate in chemical bonding mainly depends on the critical temperature owing to their inherent zwitterionic nature. Further, we found that the tin carboxylate salts produced by the reaction between Glu and SAC305 could function as catalysts for the etherification of epoxides and alcohols. This led to the conversion of the thermoplastic network formed by thermal curing between DGEBF and Glu into a highly crosslinked network that imparted excellent impact reliability. To the best of our knowledge, this is the first report in the literature on the mechanism of the thermochemical reaction between DGEBF, Glu, and SAC305 that also demonstrates the potential use of their combination as an ESP with relatively low outgas, good solderability, and epoxy encapsulation around solder joints compared with those of the conventional solder pastes. We believe that our findings can be applied to a wide range of chemical formulations consisting of epoxides, amino acids, and solder powder for electrical joining, applicable to fine-pitch soldering such as in the fabrication of mini-LEDs and small-sized MLCCs.

## Data Availability

The data presented in this study are available on request from the corresponding author.

## Supplementary Materials

The following are available online at https://www.mdpi.com/2073-4360/13/6/957/s1, Video S1: High temperature behavior of c-EG, Video S2: High temperature behavior of c-EGS-1, Video S3: Ball drop test of c-EG, Video S4: Ball drop test of c-EGS-1, Video S5: Thermal reflow test of EGS-80, Video S6: Thermal reflow test of solder paste.
